# PINK1 mediates neuronal survival in monkey

**DOI:** 10.1007/s13238-021-00889-w

**Published:** 2021-11-23

**Authors:** Zhiyu Sun, Jiangyu Ye, Junying Yuan

**Affiliations:** Interdisciplinary Research Center on Biology and Chemistry, Shanghai Institute of Organic Chemistry, Chinese Academy of Sciences, Shanghai, 201210 China

Parkinson’s disease (PD) is one of the most common neurodegenerative diseases with progressive motor dysfunction and pathologically characterized by the degeneration of dopaminergic neurons in the substantia nigra of the midbrain and the development of neuronal Lewy bodies. Autosomal recessive mutations in the gene PINK1 leads to inherited idiopathic PD. PINK1 is known to regulate PARKIN translocation upon mitochondrial damage which drives their removal via selective autophagy, a process known as mitophagy (Pickrell and Youle 2015; Cummins and Gotz 2018). While mitochondrial defects had been noted in postmortem studies of patient brain samples with PD, there is a continuing debate as how mutations in PINK1 may lead to selective neuronal death in the substantia nigra.

At least in part due to the difficulty in demonstrating neurodegenerative phenotypes in PINK1 knockout mouse models, the evidence that supports the contribution of defective mitophagy to PINK1-related parkinsonian syndromes is still circumstantial. Monkey models may provide new insights into human diseases. Yang et al. have previously reported a monkey model with PINK1 deficiency generated by CRISPR-Cas9 (Yang et al. 2019). This CRISPR-based PINK1 deficient monkey model, which exhibits obvious reduction in the protein levels of PINK1, demonstrates robust neurodegeneration in both substantia nigra and cortex, more extensive than what is found in late-onset familial PD patients with homozygous point mutation in PINK1 that can be explained by the nature of a partial loss-of-function. In this current study, Yang et al. used AAV-gRNA based strategy by stereotaxic injection to achieve local PINK1 knockdown in prefrontal cortex and substantia nigra in adult rhesus monkeys. Strikingly, knockdown of PINK1 leads to substantial loss of neurons and motor dysfunction with activation of caspase-3 in monkey. Demonstrating neuronal loss by PINK1 knockout or knockdown in monkey is exciting as it provides an important tool for investigating the mechanism of neuronal cell death in human PD. Using EM analysis, Yang et al. demonstrate degenerating neurons in the brains of adult PINK1 knockdown monkey with increased lysosomal and phagocytic vacuoles but without clear evidence of mitochondrial abnormality. This observation questions if defects in mitophagy, as implicated by many previous studies, are responsible for neurodegeneration in human PD.

While most previous studies focused on a 70 kDa PINK1 protein product, which can be stabilized by mitochondrial damage (Okatsu et al. 2012), Yang et al. found a dominant cytosolic 55 kDa PINK1 protein product which was reduced in CRISPR-based PINK1 knockout monkey, suggesting that this 55 kDa PINK1 protein is a major product from the PINK1 locus in the brains. Yang et al. found that this 55 kDa PINK1 was expressed at the undetectable level in control rodent brains, which may explain the lack of phenotype in PINK1 knockout mice. This 55 kDa PINK1 protein was abundantly present in human brains, but not in peripheral tissues, such as liver, heart, muscle, intestine or kidney. Interestingly, this 55 kDa PINK1 protein was abundantly present in the substantia nigra, the brain area most vulnerable in PD (Yang et al. 2021). The relationship between the 70 kDa and 55 kDa PINK1 products should be explored in future.

Yang et al. conducted further biochemical characterization of monkey brain proteins with PINK1 knockdown. The authors found a dramatic loss of synaptic proteins, PSD95, SNAP25, crmp2 and synaptophysin in the fetal PINK1 knockout monkey model. In contrast, no changes in the mitochondrial complex proteins, VDAC1 or Tom20, or metabolites were found in this PINK1 knockout monkey. The striking preferential loss of synaptic proteins in fetal monkey PINK1 knockout brains suggest the role of PINK1 in developing synapses. While deposits of fibrillary α-synuclein aggregates, known as Lewy bodies and Lewy neurites, are primarily presynaptic in nature, defective postsynaptic mechanisms involving NMDA receptors in striatal spiny neurons have also been implicated in PD (Picconi et al. 2012).

Yang et al. showed that PINK1 knockdown in monkey brains leads to substantial reduction in the phosphorylation of many proteins, including many that are involved in neuronal function and survival. Further studies are needed to elucidate how PINK1 may be involved in regulating the phosphorylation of so many proteins. This study challenges the idea that PINK1 is primarily involved in maintaining mitochondrial homeostasis per se. At the minimum, Yang et al. provides compelling reasons to examine the mitophagy dependent and independent role of PINK1 in Parkinson’s disease.
