# Correction to: PINK1 mediates neuronal survival in monkey

**DOI:** 10.1007/s13238-021-00899-8

**Published:** 2021-12-30

**Authors:** Ziyu Sun, Jianyu Ye, Junying Yuan

**Affiliations:** Interdisciplinary Research Center on Biology and Chemistry, Shanghai Institute of Organic Chemistry, Chinese Academy of Sciences, Shanghai, 201210 China

## Correction to: Protein Cell 10.1007/s13238-021-00889-w

In the Original version of the article, the first two author names were incorrect.

The Correct names are given below:

Ziyu Sun, Jianyu Ye.

